# 00006 Yield of routine PET/CT surveillance imaging after primary surgical treatment for asymptomatic patients with high-risk stage II/III melanoma

**DOI:** 10.1017/cts.2021.691

**Published:** 2021-03-30

**Authors:** Zachary J. Jaeger, Rachel E. Reed, Abigail R. Barker, Joyce C. Mhlanga, Lynn A. Cornelius, Ryan C. Fields

**Affiliations:** All at Washington University School of Medicine

## Abstract

ABSTRACT IMPACT: The results of this research may influence NCCN guidelines on PET/CT surveillance for this population of patients for whom the guidelines are currently vague. OBJECTIVES/GOALS: We aim to quantify and describe the yield of surveillance PET/CT for detecting asymptomatic recurrence of melanoma after primary surgical treatment for stages IIB, IIC, and IIIA. Our goal is to provide evidence to inform appropriate guidelines for scheduling surveillance PET/CT for this population. METHODS/STUDY POPULATION: This is a retrospective study of patients who have undergone a PET/CT at Barnes-Jewish Hospital. Data will be collected in our Research Electronic Data Capture (REDCap) database. The sample size is 158. Data analysis will be explanatory for the yield of imaging and description of false positives and additional unnecessary workup. Survival endpoints will be reported and multivariate analysis with subgroups will be performed for predictors of PET/CT results. Cost-efficiency analysis will be conducted in collaboration with the Center for Health Economics and Policy (CHEP), with emphasis on a patient-oriented perspective. RESULTS/ANTICIPATED RESULTS: To date, we have collected data for 158 patients, with approximately equal numbers of each stage (56 IIB, 54 IIC, 48 IIIA). Due to lack of clear guidelines for this population, there is significant variation of imaging schedules and results between similar patients or groups of patients. This makes it difficult to anticipate results. Based on clinical experience, literature review, and preliminary data, we may anticipate lower yield of routine PET/CT for the detection of asymptomatic recurrence, with frequent false negatives and occasional false positives, in addition to detection and further workup of benign processes. DISCUSSION/SIGNIFICANCE OF FINDINGS: The high-risk stages IIB, IIC, and IIIA currently occupy a gray area where surveillance PET/CT remains controversial. For this group of patients, it is important to weigh the benefits of early detection and risks of false positives, unnecessary workup and anxiety, and cost.

